# High Rates of Abnormal Glucose Metabolism Detected by 75 g Oral Glucose Tolerance Test in Major Psychiatric Patients with Normal HbA1c and Fasting Glucose Levels

**DOI:** 10.3390/nu17040613

**Published:** 2025-02-08

**Authors:** Sumiko Yoshida, Emiko Aizawa, Naoko Ishihara, Kotaro Hattori, Kazuhiko Segawa, Hiroshi Kunugi

**Affiliations:** 1 Department of Psychiatric Rehabilitation, National Center of Neurology and Psychiatry Hospital, Tokyo 187-8551, Japan; 2 Mood Disorder Center for Advanced Therapy, National Center of Neurology and Psychiatry Hospital, Tokyo 187-8551, Japan; ishihara@ncnp.go.jp; 3 Medical Genome Center, National Center of Neurology and Psychiatry, Tokyo 187-8502, Japan; hattori@ncnp.go.jp; 4 Department of Mental Disorder Research, National Institute of Neuroscience, National Center of Neurology and Psychiatry, Tokyo 187-8502, Japan; e-aizawa@sendai-shirayuri.ac.jp; 5 Faculty of Human Sciences, Sendai Shirayuri Women’s University, Sendai 981-3107, Japan; 6 Department of General Medicine, National Center of Neurology and Psychiatry Hospital, Tokyo 187-8551, Japan; ksegawa@ncnp.go.jp; 7 Department of Psychiatry, Teikyo University School of Medicine, Tokyo 173-8605, Japan

**Keywords:** bipolar disorder, glucose metabolism, major depressive disorder, diabetes, 75 g oral glucose tolerance test, schizophrenia

## Abstract

**Objectives**: Comorbid diabetes is an important factor in the treatment of major psychiatric disorders. However, a substantial proportion of diabetic patients remain undetected by routine diabetic indices such as blood glucose and HbA1c. This study tried to estimate rates of such unidentified diabetic or prediabetic patients by using a 75 g oral glucose tolerance test (OGTT). **Methods**: Participants in the test were 25 patients with major depressive disorder (MDD), 28 patients with bipolar disorder (BP), 26 patients with schizophrenia, and 28 psychiatrically normal controls. They were all Japanese, and showed non-diabetic levels of blood glucose (<126 mg/dL) and HbA1c (<6.0%). **Results**: Relatively high rates of psychiatric patients showed diabetes mellites (DM)-type abnormality (32% of MDD, 21.4% of BP and 42.3% of schizophrenia v. 10.7% of controls). The difference in the rates between schizophrenia and control groups was statistically significant (*p* = 0.008). When abnormal glucose metabolism was defined as a prediabetic state (either normal high glycemia, impaired fast glycemia or impaired glucose tolerance) or DM type in OGTT, it was more frequently seen in the psychiatric patients than in controls (64% of MDD, 46.4% of BP and 46.2% of schizophrenia v. 35.7% of controls). Individuals with DM type showed higher HbA1c values compared with those with normal (*p* < 0.001) and prediabetic (*p* = 0.021) states. **Conclusions**: The results suggest that relatively high proportions of patients with a major psychiatric disorder remain undetected by routine indices for abnormal glucose metabolism, indicating the importance of OGTT even if the patients showed non-diabetic levels in blood glucose or HbA1c.

## 1. Introduction

A number of previous studies have shown that metabolic syndrome and/or diabetes mellitus (DM) are associated with major psychiatric disorders such as major depressive disorder (MDD) [1,2], bipolar disorder (BP) [3,4,5,6], and schizophrenia (Sz) [7,8,9,10,11,12,13]. A recent umbrella review on meta-analytic studies reported a higher prevalence of DM in patients with depression (9%), BP (11%), and Sz (10%) relative to the general population (6 to 9%) [14]. In Japan, Yoshida et al. [15] reported that approximately one third (36.4%) of diabetic patients had depressive symptoms and 7.9% were diagnosed as MDD. Further, a diabetic complication, specifically neuropathy, was independently associated with depression in DM patients, which raised the possibility that DM shares some biological mechanisms with MDD.

Although the 75 g oral glucose tolerance test (OGTT) is the gold standard to detect abnormal glucose metabolism, this method is time- and manpower-consuming. It is also hampered by low reproducibility [16]. Therefore, a diagnosis of diabetes has often been made based on laboratory index tests such as blood glucose (random/fasting) and hemoglobin A1c (HbA1c) in daily clinical practice. However, the sensitivity of HbA1c is inadequate (e.g., 50% according to a recent meta-analysis [17], indicating that a substantial proportion of people with diabetes or its pre-stage are undetected by the index tests. For example, a study from the Netherlands conducted OGTT in 200 patients with Sz or schizoaffective disorder and reported the prevalence of DM as 14.5%, of which 6.5% was newly diagnosed [18]. Still, there is a dearth of research using OGTT in psychiatric patients.

In this study, we tried to elucidate the rates of such individuals with abnormal glucose metabolism by using 75 g OGTT in major psychiatric patients who showed normal blood fasting glucose and HbA1c levels.

## 2. Materials and Methods

### 2.1. Participants

Participants were 25 patients with MDD (mean age 40.8 ± 12.0 years, 11 females), 28 patients with BP (41.0 ± 8.8 years, 17 females), 26 patients with Sz (38.3 ± 9.3, 13 females), and 28 psychiatrically healthy controls (41.6 ± 11.1 years, 16 females), recruited from the outpatient clinic at the National Center of Neurology and Psychiatry Hospital and the local community through our website and local magazine advertisements. The participants were all Japanese. The psychiatric diagnosis was made by board-certificated psychiatrists according to the *Diagnostic Statistical Manual of Mental Disorders* 4th edition [19] after a Mini-International Neuropsychiatric Interview [20,21], additional unstructured interview, and review of medical records. The participants had no apparent history of DM, and all individuals showed a fasting glucose concentration of <126 mg/dL and HbA1c of <6.0%. The psychiatric patients were a mixture of acute/chronic and remitted/non-remitted patients. The study protocol was approved by the ethics committee at the National Center of Neurology and Psychiatry (A2012-035) and was performed in accordance with the Declaration of Helsinki [22]. The procedures were fully explained, and written informed consent was obtained from each participant.

### 2.2. Measurement

We measured height, body weight, body mass index (BMI), abdominal circumference, HbA1c (National Glycohemoglobin Standardization Program), serum lipids (total cholesterol; T-Cho and triglycerides; TG), renal function (urea nitrogen; UN and creatinine clearance; CRE), and liver function (aspartate aminotransferase; AST, alanine aminotransferase; ALT and γ-glutamyltransferase; γ-GT). These biochemical markers were measured by SRL co., Tokyo, Japan. Information on psychiatric medication was obtained from medical records and daily doses of antipsychotics and antidepressants were converted to equivalent doses of chlorpromazine and imipramine, respectively, according to the published guideline [23].

### 2.3. Classification of Glucose Metabolism Using 75 g OGTT

The classification was carried out based on the blood glucose concentration before 75 g OGTT and 30, 60, 90, and 120 min after 75 g OGTT: (I) DM type: More than 200 mg/dL at 120 min after glucose load. (II) Borderline type (impaired glucose tolerance (IGT)): 140–199 mg/dL at 120 min after glucose load. (III) Impaired fast glycaemia (IFG): 110–125 mg/dL before glucose load. (IV) Sub-borderline type: More than 180 mg/dL at 60 min after glucose load. (V) Fasting normal hyperglycemia: A total of 100–109 mg/dL before glucose load. (VI) Normal type: Less than 100 mg/dL before glucose load and less than 140 mg/dL at 120 min after glucose load. In this study, II–V types were considered to have prediabetic abnormal glucose metabolism. This classification was made according to the classification and diagnostic criteria of DM of the Japan Diabetes Society [24] by referring to several international criteria, including those of the American Diabetic Association [16].

### 2.4. Statistical Analysis

To compare differences in characteristics across normal control and psychiatric diagnostic groups, the non-parametric Kruskal–Wallis test (post hoc comparisons with Bonferroni correction) was used because many parameters deviated from the normal distribution according to the Shapiro–Wilk test. The χ^2^ test was used to compare the rates of abnormal glucose metabolism classifications. All statistical analyses were performed using SPSS for Windows, version 18. A value of *p* < 0.05 was considered statistically significant.

## 3. Results

### 3.1. Comparison of Characteristics

Characteristics of the participants are presented in Table 1. There was no significant difference between any psychiatric diagnostic group and controls on demographic (gender and age) or physical characteristics (height, body weight, BMI, and abdominal circumference), or in HbA1c, serum lipids (T-Cho and TG), CRE, or liver functions (AST, ALT, and γ-GT).

### 3.2. Medication

There were 22 “drug-free” patients (7 MDD, 11 BP, and 4 Sz) who had not taken any psychotropic medication for 3 months or more before the OGTT. All Sz patients and half of the BP patients took second-generation antipsychotics, except the medication-free patients.

### 3.3. Oral Glucose Tolerance Test

**Figure 1:**
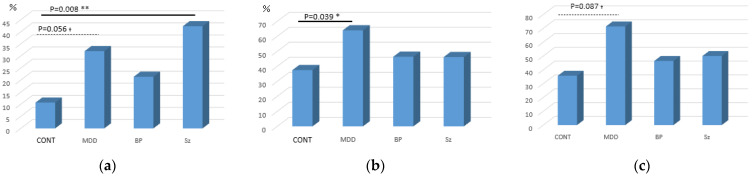
Frequencies of glucose metabolism abnormalities: (**a**) DM type; (**b**) abnormal glucose metabolism in total participants (DM + prediabetic); (**c**) drug-free participants. Solid and dotted lines indicate *p*-values of significant and non-significant trend-level differences, respectively. *: *p* < 0.05; **: *p* < 0.01, ^†^: *p* < 0.10.

Figure 1 shows the rates of glucose metabolism abnormalities assessed with 75 g OGTT in psychiatric patients and controls. Three out of the healthy control group (10.7%), eight out of the MDD group (32%), six out of the BP group (21.4%), and eleven out of the Sz group (42.3%) showed DM type, respectively (Figure 1a). Patients with MDD showed a higher frequency of DM type abnormality than controls at a trend level (χ^2^ = 3.64, degree of freedom [*df*] = 1, Cramer’s V = 0.26, *p* = 0.056), and there was a significant difference in the frequency of DM type abnormality between patients with Sz and controls (χ^2^ = 6.1, *df* = 1, Cramer’s V = 0.36, *p* = 0.008). There was no significant difference in frequency of DM type abnormality between patients with BP and controls.

When “abnormal glucose metabolism” was defined as having either normal high glycemia, IFG, IGT, and/or DM type in OGTT, 16 patients with MDD (64%), 13 patients with BP (46.4%), 12 patients with Sz (46.2%), and 10 controls (35.7%) showed an abnormal glucose metabolism. When these rates in the psychiatric groups were compared with those of healthy controls, there was a statistical difference in the MDD group (χ^2^ = 4.23, *df* = 1, Cramer’s V = 0.28, *p* = 0.039). These abnormalities may have arisen due to psychiatric medication. However, even when the participants were restricted to medication-free patients, five out of seven MDD (71.4%), four out of eleven BP (36.4%), and two out of four Sz (50%) groups showed abnormal glucose metabolism. Although a comparison between each of these rates with those of controls did not reach statistical significance, the medication-free MDD patients had a higher frequency of “abnormal glucose metabolism” than healthy controls at a trend level (χ^2^ = 2.92, *df* = 1, Cramer’s V = 0.29, *p* = 0.087). We then examined the possible relationships between medication and abnormal glucose metabolism. We compared antidepressant and antipsychotic doses (imipramine and chlorpromazine equivalent, respectively) across DM type, prediabetic state, and normal groups in MDD and Sz patients. We found no significant difference in antidepressant or antipsychotic dose across the three groups for either the MDD (*p* = 0.36) or Sz (*p* = 0.64) group.

**Figure 2:**
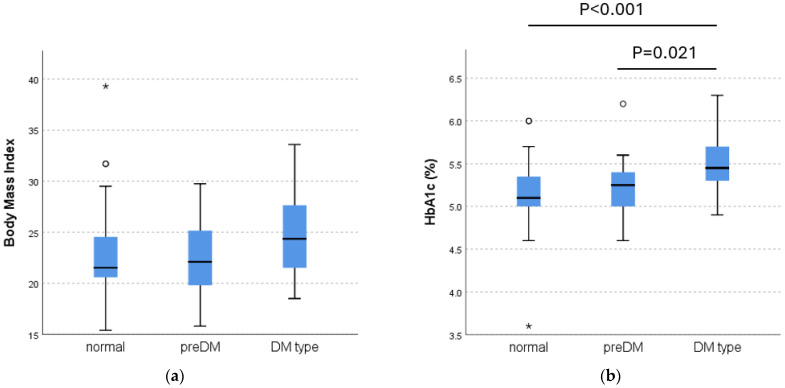
BMI and HbA1c among normal, prediabetic, and DM-type groups in the total participants. (**a**) Body mass index (BMI). (**b**) HbA1c. Box plots show median, interquartile, and 95th percentile ranges. Circles and an asterisk show outliers.

Next, we compared BMI and HbA1c among the three groups of normal, prediabetic, and DM type groups in the total participants (Figure 2). There was no significant difference in BMI across the three groups, although the difference approached statistical significance (*p* = 0.098) (Figure 2a). However, there was a significant difference in HbA1c across the three groups (*p* < 0.001). Post hoc analysis with Bonferroni correction showed a significant difference between DM type and normal groups (*p* < 0.001) and between DM type and prediabetic groups (*p* = 0.021), but not between prediabetic and normal groups (*p* = 1.0).

## 4. Discussion

This study investigated glucose metabolism in patients with a major psychiatric disorder and healthy controls who showed normal values of fasting glucose and HbA1c by using 75 g OGTT. To our knowledge, this is the first study to have examined the three major psychiatric diseases and a healthy comparison group for OGTT. We found substantially higher rates of DM type and abnormal glucose metabolism in the patient groups compared with the controls, particularly in those with Sz and those with MDD. In addition, we found higher HbA1c in DM-type groups compared with prediabetic or normal groups, indicating that individuals with a relatively higher HbA1c value (particularly >5.5, according to Figure 2) should be examined with OGTT.

### 4.1. Schizophrenia

It has long been reported, since the pre=antipsychotic era, that patients with Sz have an increased risk for DM [7,8,9]. Although it is well known that some of the second-generation antipsychotics, such as olanzapine and clozapine, impair glucose metabolism (reviewed in [25,26]), a national-wide cohort study showed an increased risk for DM even in the first-generation antipsychotics era [12]. Risk factors other than antipsychotic medication include unhealthy lifestyles (e.g., unhealthy food habits and sedentary lifestyle), limited access to medical care, and genetic factors including a family history of DM in patients with Sz [10,11,13,27]. It is also possible that Sz and type 2 DM share common genetic risk factors [28,29].

Regarding studies in Japan, a previous nationwide study in psychiatric patients showed a relatively high prevalence of DM (8.6%) among discharged patients with Sz [30]. In this study, information on a diagnosis of DM was based on medical records (i.e., a physician’s diagnosis based on unknown reasons). Sugai et al. [31] conducted a Japanese nationwide study in a total of 7655 outpatients and 15,461 inpatients with Sz and reported that the prevalence of DM (defined as fasting glucose of 126 mg/dL or more) was 16.1% and 7.1%, respectively. A main reason for the discrepancy between out- and inpatients might be that a substantial proportion of Japanese inpatients had had their diet managed by a dietician at their hospital for a long time. Ono et al. [32] administered 75 g OGTT for 258 inpatients with Sz with normal fasting glucose levels, and reported that 1.3% were newly diagnosed with diabetes and 17.3% had impaired glucose tolerance. However, our prevalence of DM type (42.3%) in Sz outpatients was much higher compared with these results. These discrepancies might be due, at least in part, to the method (medical records or fasting glucose level vs. 75 g OGTT) and participants (inpatient vs. outpatients). The observed high prevalence might be worthy of attention since there is a dearth of information on outpatients with Sz assessed with OGTT in Japan.

Notably, antidiabetic pharmacotherapy has been suggested to be effective in antipsychotic-induced weight gain, as well as glucose metabolism. A meta-analysis of 12 published studies (a total of 743 patients) found that in patients treated with antipsychotics, metformin treatment resulted in significantly reduced BMI and metabolic parameters (insulin resistance index) compared to placebo [33]. More recent clinical trials suggest that glucagon-like peptide-1 (GLP-1) receptor agonists are effective on body weight as well [34]. Interestingly, a recent open-label clinical trial suggested that metformin improves cognitive impairment in patients with Sz, which was associated with the enhanced functional connectivity of dorsolateral prefrontal cortex [35]. Preclinical studies have demonstrated that GLP-1 receptor agonists improve cognition [36]. A systematic review and meta-analysis demonstrated that non-pharmacological interventions on type 2 DM also significantly reduce psychiatric symptoms in severe mental illnesses [37]. Moreover, meta-analytic studies have shown that exercise therapy is effective on psychiatric symptoms, cognitive symptoms, and overall quality of life in Sz patients [38,39]. In our related study, we reported that cognitive function in patients with Sz is correlated positively with handgrip strength and negatively with BMI [40].

### 4.2. Major Depressive Disorder

Patients with MDD had an increased risk for abnormal glucose metabolism including type 2 DM [1,2], which is consistent with our results. In a Japanese longitudinal study, wherein 2764 men were followed up for 8 years, those who had depressive symptoms had a 2.3-fold higher risk of developing DM in the future than those who did not [41]. The relatively high risk may have come about because physical-activity-enhancing interventions have not been well established in the management of depressive symptoms in Japan. Previously, we performed an Internet cross-sectional survey to obtain data on 11,876 Japanese individuals, including 1000 with a history of depression. We found that a diagnosis of DM was 1.5 times more common in individuals with a history of depression than those without such a history [42]. Further, a meta-analysis published in 2006 showed a significantly increased prevalence of depression in patients with type 2 diabetes compared with those without (17.6 vs. 9.8%) [43]. Therefore, the bidirectional association between type 2 DM and depression has been established.

A possible mechanism might be that stress triggers both depression and DM. Psychological pressures affect the autonomic nervous system and alter the hypothalamic–pituitary–adrenal axis, which leads to depressive disorder [44,45,46,47] and metabolic abnormalities including visceral fat accumulation and insulin resistance [48]. In fact, stressful life events and their accumulation increase the risk of developing not only depression [49], but also metabolic syndrome, including obesity and insulin resistance [50].

Another possible mechanism is the effect of antidepressants. It is known that antidepressants such as mirtazapine, amitriptyline, and paroxetine have significant weight gain effects in maintenance therapy (reviewed in [51]), which may confer the risk of developing DM or a prediabetic state. In the review by Hennings et al. [52], however, selective serotonin reuptake inhibitors had a beneficial effect on glucose homeostasis, although other antidepressants (noradrenergic and possibly dual active substances) may deteriorate glucose tolerance. Therefore, the effects of antidepressants on glucose metabolism may be mixed. It is possible that a portion of our patients were affected by antidepressants. Nevertheless, 71.4% of medication-free patients with MDD showed abnormal glucose metabolism in our study, indicating that clinicians should be cautious about the risk of developing DM and that lifestyle factors including diet should be reevaluated in the treatment of MDD [53].

In depressed patients with diabetes and possibly those with prediabetes, antidiabetic drugs such as metformin and GLP-1 receptor agonists such as liraglutide seem to be effective in reducing depressive symptoms and cognitive impairments [54,55,56]. For example, a clinical trial in China, examining the effect of metformin in patients with MDD and type 2 DM, reported that metformin was associated with improved depression scores together with improved cognition, compared with a placebo [57]. In addition, exercise has now been established as an effective strategy to both prevent and improve depression [58,59,60]. Education by dieticians on daily diet should also be considered because excessive consumption of a Western diet, particularly involving ultra-processed food, increases the risk of both DM and depression [61,62].

### 4.3. Bipolar Disorder

Patients with BP also have an increased risk of developing metabolic syndrome, type 2 DM, and cardiovascular disease when compared with the general population [3,4,5,6], although contradictive negative results have also been reported [63]. Since our BP patients did not significantly differ in the rate of DM type or that of abnormal glucose metabolism from those of healthy controls, we could not confirm the results of the above reports [3,4,5,6]. However, given that the rates of these abnormalities showed higher values (albeit non-significant ones) in the BP group compared to the controls, our failure to obtain a significant difference might be due to the inadequate statistical power of the small sample size. To our knowledge, there is no study that performed OGTT in Japanese BP subjects. Further studies with a larger number of BP patients are warranted. Nonetheless, comorbid DM and a prediabetic state are associated with poorer clinical outcomes. For example, Leopold et al. [64] performed OGTT in 85 euthymic outpatients with bipolar disorders in Germany and found DM and pre-diabetic abnormalities in 7% and 27% of their patients, respectively. The group of patients with abnormalities in their glucose metabolism had significantly lower quality of life and global functioning.

### 4.4. Limitations

Our study was limited by the small sample size, which is subject to type I and II errors. Therefore, the reliability and generalizability of the findings are constrained. It is essential to conduct further studies with larger sample sizes and diverse populations to address these limitations. Concerning the rates of prediabetic and DM-type abnormalities, we did not make corrections for multiple testing. Since there were three psychiatric diagnostic groups, the critical *p*-value would have been 0.016 (0.05/3) if the Bonferroni method was applied. The observed higher rate of prediabetic or DM-type abnormality in MDD patients (uncorrected *p* = 0.039) would have become non-significant.

The majority of our psychiatric patients received psychotropic medication, which is likely to have affected the results, as described above. In particular, the number of drug-free patients was quite small, which requires further investigations in a larger sample size of drug-free patients. In addition, our psychiatric patients were a mixture of acute/chronic and remitted/non-remitted individuals. It is possible that such illness stages may have affected the results of OGTT. In this study, genetic (family history of DM) and lifestyle factors such as exercise and diet were not taken into account in the analysis. We hope that we will be able to address these factors in future studies. Finally, our study might have recruited participants who had a suspicion about metabolic syndrome and/or DM, which may have resulted in exaggerated prevalence in our patients.

## 5. Conclusions

We examined glucose metabolism abnormalities by using 75 g OGTT in patients with a major psychiatric disorder and healthy control participants who apparently showed normal HbA1c and fasting glucose levels. The patients with Sz had a higher prevalence of DM type compared with healthy controls. Regarding abnormal glucose metabolism, there were significant differences in patients with MDD compared with healthy controls. Furthermore, 71.4% of medication-free patients with MDD showed abnormal glucose metabolism. Our results indicate the importance of 75 g OGTT in patients with a major psychiatric disorder (Sz or MDD, in particular) to detect potential abnormalities in glucose metabolism even if patients appear to be “normal” as regards routine diabetic indices such as blood glucose and HbA1c. Although further studies in more diverse populations are needed, the use of OGTT in psychiatric care should be emphasized. The literature suggests that interventions on lifestyle habits such as diet, exercise, and stress coping and antidiabetic drugs are effective for not only glucose metabolism but also psychiatric symptoms including cognitive functions and overall quality of life in patients with a major psychiatric disorder. Intervention studies are warranted to demonstrate this possibility.

## Figures and Tables

**Table 1:** Characteristic variables of the participants.

	Healthy Control	MDD	BP	Sz
N = 107(M 50/F 57)	28 (12/16)	25 (14/11)	28 (11/17)	26 (13/13)
Age (years)	41.6 ± 11.1	40.8 ± 12.0	41.0 ± 8.8	38.3 ± 9.3
Education (years)	15.8 ± 2.5	15.2 ± 2.6	15.7 ± 2.3	14.1 ± 3.0
Height (cm)	163.5 ± 8.9	165.6 ± 9.5	163.1 ± 8.5	166.1 ± 8.3
Weight (kg)	60.9 ± 11.7	62.2 ± 11.8	62.1 ± 14.5	68.4 ± 17.8
Body mass index: BMI	22.7 ± 3.2	22.5 ± 2.9	23.3 ± 4.5	24.7 ± 5.3
Abdominal circumference (cm)	80.9 ± 10.0	80.9 ± 8.7	82.0 ± 11.2	86.0 ± 14.5
Fasting glucose (mg/dL)	93.6 ± 8.3	95.3 ± 9.5	92.1 ± 8.5	94.6 ± 5.7
HbA1c (NGSP: %)	5.2 ± 0.5	5.3 ± 0.4	5.3 ± 0.4	5.3 ± 0.3
AST (IU/L)	22.5 ± 10.5	23.3 ± 15.4	22.2 ± 8.3	22.8 ± 11.8
ALT (IU/L)	18.6 ± 9.7	27.8 ± 37.6	24.7 ± 16.5	29.2 ± 24.1
γGTP (IU/L)	27.3 ± 28.3	33.4 ± 32.7	28.3 ± 26.1	31.6 ± 27.5
TG (mg/dL)	104.4 ± 99.5	116.6 ± 86.9	129.0 ± 89.0	144.0 ± 165.0
T-Cho (mg/dL)	204.4 ± 44.4	213.2 ± 29.4	199.8 ± 32.1	184.1 ± 36.9
BUN	12.1 ± 2.8	13.5 ± 3.6	11.1 ± 4.1	11.5 ± 4.3
CRE	0.67 ± 0.02	0.74 ± 0.03	0.70 ± 0.03	0.73 ± 0.03
Antidepressant (mg) (imipramine equivalents)	0.00	100.9 ± 102.8	41.0 ± 64.8	9.1 ± 31.1
Antipsychotics (mg) (chlorpromazine equivalents)	0.00	21.5 ± 55.4	84.1 ± 163.6	447.1 ± 511.3
Psychiatric medication-free N	28	7	11	4

(Mean ± SD).

## Data Availability

The original contributions presented in this study are included in the article. Further inquiries should be directed to the corresponding authors. The data are not publicly available due to ethical reasons.

## Abbreviations

The following abbreviations are used in this manuscript:

ALT	alanine aminotransferase
AST	aspartate aminotransferase
BMI	body mass index
BP	bipolar disorder
CRE	creatinine clearance
DM	diabetes mellitus
DSM-IV	*Diagnostic and Statistical Manual of Mental Disorders*, 4th edition
γ-GT	γ-glutamyltransferase
GLP-1	glucagon-like peptide-1
HbA1c	hemoglobin A1c
MDD	major depressive disorder
OGTT	oral glucose tolerance test
Sz	schizophrenia
T-Cho	total cholesterol
TG	triglycerides
UN	urea nitrogen
